# Dr. Waldon on Scarlatina

**Published:** 1811-01

**Authors:** John Waldon

**Affiliations:** Bodmin, Cornwall


*To the Editors of the Medical and Physical Journal.*

GENTLEMEN,

IN a Number of your very useful Journal published three or four years since, I took the liberty of suggesting a mode of treatment in Scarlatina Anginosa Maligna, which was happily attended with the most complete success.

I cannot at this distant period charge my recollection with the circumstances, either local or general, under which the disease made its appearance, or by which its fatality might have been influenced, not having that Number of the Medical and Physical Journal immediately within my reach, but that the disease in a very great and alarming number of instances ran its fatal course in a very short period, notwithstanding both the most rigorous antiphlogistic and antiseptic treatment had been had recourse to, in succession, without the least apparent benefit whatever. By a reference to the paper alluded to, it will be seen that, as soon as the carbonic acid gas was liberated in the pharynx, œsophagus, and stomach, from the supercarbonated kali, its antiseptic powers were immediately apparent in almost every instance. In the hope that, should Mr. Goodwin be induced to make trial of the above treatment, he will be equally successful with myself in this truly alarming disease, I remain, Gentlemen,

Your most obedient humble servant,

JOHN WALDON, M.D.

*Bodmin, Cornwall, Dec. 9th,* 1810.

